# Integrative Taxonomy of *Tereancistrum* spp. (Monopisthocotyla: Dactylogyridae) Parasites of the Gills of Freshwater Fishes from the Caatinga Domain, Brazil

**DOI:** 10.3390/pathogens14050467

**Published:** 2025-05-10

**Authors:** Priscilla de Oliveira Fadel Yamada, Wallas Benevides Barbosa de Sousa, Mariana Bertholdi Ebert, Maria Fernanda Barros Gouveia Diniz, Marcos Tavares-Dias, Reinaldo José da Silva, Fabio Hideki Yamada

**Affiliations:** 1 Programa de Pós-Graduação em Diversidade Tropical (PPGBio), Universidade Federal do Amapá (UNIFAP), Macapá 68903-419, AP, Brazil; marcos.tavares@embrapa.br; 2 Programa de Pós-Graduação em Diversidade Biológica e Recursos Naturais (PPGDR), Universidade Regional do Cariri (URCA), Crato 63105-000, CE, Brazil; wallasbenevides@gmail.com (W.B.B.d.S.); mbe.bio@gmail.com (M.B.E.); fernanda.gouveia@urca.br (M.F.B.G.D.); fabio.yamada@urca.br (F.H.Y.); 3 Embrapa Amapá, Macapá 68903-419, AP, Brazil; 4 Instituto de Biociências, Departamento de Biodiversidade e Bioestatística, Universidade Estadual Paulista (UNESP), Botucatu 18618-689, SP, Brazil; reinaldo.silva@unesp.br

**Keywords:** new species, Prochilodontidae, Anostomidae, Neotropical region, LSU rDNA, molecular data, phylogenetic analyses

## Abstract

*Tereancistrum* is a common genus of Neotropical monopisthocotylans; however, information on its diversity and phylogeny remains limited. In this study, we describe four new species of *Tereancistrum* parasitizing the gills of *Prochilodus brevis* (Characiformes: Prochilodontidae) from a weir in the state of Ceará, Brazil. *Tereancistrum spiralocirrum* n. sp. and *Tereancistrum scleritelongatum* n. sp. are characterized by a dextro-ventral vaginal pore and a Y-shaped dorsal bar. Notably, *Tereancistrum spiralocirrum* n. sp. is the first species in the genus to possess a male copulatory organ (MCO) with multiple rings (16 to 18). In contrast, *Tereancistrum ancistrum* n. sp. and *Tereancistrum kritskyi* n. sp. are distinguished by a sinistral vaginal pore, a sclerotized MCO in the form of a coiled tube with slightly more than one clockwise ring, and an accessory piece that is non-articulated with the base of the MCO. However, *Tereancistrum ancistrum* n. sp. is unique in lacking a dorsal bar. Sequences of the LSU rDNA obtained from seven species of *Tereancistrum* parasitizing *P. brevis* and *Leporinus piau*, along with published sequences of other Dactylogyridae members, were included in the molecular analyses. Phylogenetic reconstructions supported the monophyly of *Tereancistrum*.

## 1. Introduction

The Neotropical region harbors the richest freshwater fish fauna in the world [1]. It is estimated that there are more than 6200 species [2], with over 3600 occurring in Brazilian waters [1]. Among this immense diversity, fishes from the families Anostomidae and Prochilodontidae stand out due to their wide distribution across the Neotropical region and their significant ecological and economic roles [1,3,4].

Platyhelminthes of class Monopisthocotyla Brabec, Salomaki, Scholz & Kuchta, 2023 are a key component of the parasitic fauna of freshwater fishes in the neotropics, with more than 600 species reported in South America, most of which belong to the family Dactylogyridae and parasitize the gills of their hosts [5]. Currently, seven genera of monopisthocotylans are found parasitizing the gills of fishes from the families Anostomidae (*Jainus* Mizelle, Kritsky & Crane, 1968; *Urocleidoides* Mizelle & Price, 1964; *Trinibaculum* Kritsky, Thatcher & Kayton, 1980; and *Tereancistrum* Kritsky, Thatcher & Kayton, 1980) and Prochilodontidae (*Anacanthoroides* Kritsky & Thatcher, 1976; *Apedunculata* Cuglianna, Cordeiro & Luque, 2009; *Protorhinoxenus* Domingues & Boeger, 2002; and *Tereancistrum)* in the Neotropical region [5]. Among the genera mentioned, *Tereancistrum* is the only one that parasitizes fish from both families.

*Tereancistrum* is characterized by having spathulate accessory anchor sclerites associated with the ventral anchors and was erected to accommodate three species of monopisthocotylans, parasites of Characiformes in three fish families: Bryconidae, Anostomidae, and Prochilodontidae [6]. Within the past years, new species have been proposed, and currently, there are 11 valid species accommodated in this genus: *Tereancistrum kerri* Kritsky, Thatcher & Kayton, 1980 (type species), *Tereancistrum arcuatus* Cohen, Kohn & Boeger, 2012, and *Tereancistrum campanum* Hasuike, Scorsim, Arjona, Amaral, Damacena-Silva, Araújo, Bellay, Oliveira & Takemoto, 2025—parasites of Bryconidae fishes; *Tereancistrum flabellum* Zago, Yamada, Franceschini, Bongiovani, Yamada & Silva, 2017, *Tereancistrum paranaensis* Karling, Lopes, Takemoto & Pavanelli, 2014, and *Tereancistrum parvus* Kritsky, Thatcher & Kayton, 1980—parasites of Anostomidae fishes; and *Tereancistrum curimba* Lizama, Takemoto & Pavanelli, 2004, *Tereancistrum ornatus* Kritsky, Thatcher & Kayton, 1980, *Tereancistrum pirassununguensis* Cepeda, Ceccarelli & Luque, 2012, *Tereancistrum takemotoi* Leite, Pelegrini, Azevedo & Abdallah, 2020, and *Tereancistrum toksonum* Lizama, Takemoto & Pavanelli, 2004—parasites of Prochilodontidae fishes [6,7,8,9,10,11,12,13].

The use of molecular data (i.e., LSU rDNA and COI mtDNA) in studies on Dactylogyridae Bychowsky, 1933 parasites of freshwater fishes from the neotropical region is recent [14,15]. The inclusion of this tool, together with morphological analyses, has enabled, among other advancements, the identification of cryptic species [15], the generic relocation of species [16], and the modification of the “*incertae sedis*” Status of a species into a valid one within a genus [17]. Therefore, to better understand the relationships among monopisthocotylan species, it is necessary to use molecular analyses both in the descriptions of new species and in species already described [17,18,19,20].

In this study, we investigate the monopisthocotylans associated with the gills of *Prochilodus brevis* Steindachner, 1875 (Characiformes: Prochilodontidae) and *Leporinus piau* Fowler, 1941 (Characiformes: Anostomidae) from the Salgado River sub-basin, Caatinga domain, Brazil. Through the analyses of morphological traits and molecular data from the LSU rDNA gene, we were able to describe four new species of *Tereancistrum* and investigate the phylogenetic position of the genus within the Dactylogyridae.

## 2. Materials and Methods

### 2.1. Host Collection

Forty-one specimens of *P. brevis* (10.7–22.5 cm in standard length and 42.2–392.6 g in weight) and 35 specimens of *L. piau* (14.1–21.1 cm in standard length and 106.3–249.9 g in weight) were collected between June 2022 and July 2023. The hosts were collected from three different weirs in the Salgado River basin, state of Ceará, Brazil: Lima Campos weir, municipality of Icó (6°22′59.99″ S, 38°58′0.01″ W); Ubaldinho weir, municipality of Cedro (6°35′5.58″ S, 39°14′22.27″ W); and Rosário weir, municipality of Lavras da Mangabeira (6°53′25.97″ S, 39°4′54.02″ W). The capture and transportation of fish were authorized by SISBIO (The Biodiversity Information and Authorization System—authorization #61328-2). All animal procedures were performed in full compliance with the Ethics Committee for Animal Experimentation (CEUA #00165/2018.1) of the Regional University of Cariri—URCA. Fishes were collected using a nylon monofilament gillnet. After being captured, some specimens were individually stored in plastic bags, frozen, and transported to the laboratory. In June 2024, a new collection of fishes was conducted at Lima Campos weir, and fishes were transported alive to the laboratory and freshly necropsied for the collection of *Tereancistrum* spp. that were used for the molecular analyses.

### 2.2. Parasitological Procedures

During necropsy, the gills of the fishes were removed, placed in Petri dishes with tap water, and checked for monopisthocotylans under a stereomicroscope. Some of the specimens were mounted on permanent slides with Gray and Wess’s medium [21], and some were placed on a slide with a 0.9% saline solution while still alive, for identification, and then fixed in absolute alcohol for subsequent molecular analyses. The specimens were registered in SISGEN (Sistema Nacional de Gestão do Patrimônio Genético e do Conhecimento Tradicional Associado) according to Brazilian laws under the registration number (AE735A2).

**Figure 1:**
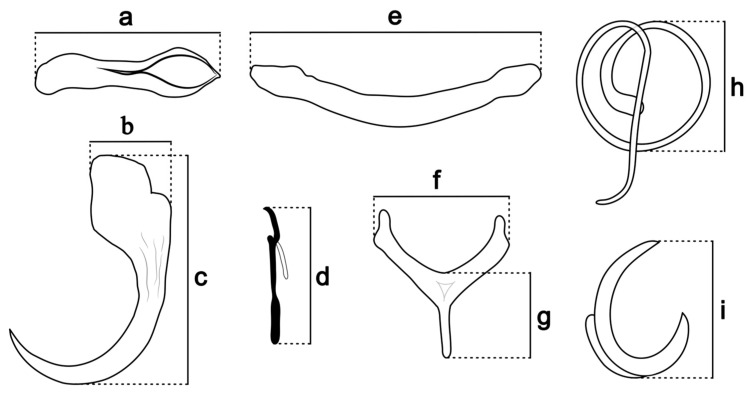
Scheme of measurements of the sclerotized structures of the copulatory complex and haptor of *Tereancistrum* spp. Accessory anchor sclerite: (**a**)—total length; ventral and dorsal anchor: (**b**)—base width, (**c**)—total length; hook: (**d**)—total length; ventral and dorsal bar: (**e**)—total length; dorsal bar with a posteromedian projection: (**f**)—total length, (**g**)—width; MCO: (**h**)—diameter of the proximal ring; accessory piece: (**i**)—total length.

The morphology and morphometry of the parasites were analyzed using an optical microscope equipped with a computerized system for image analysis with phase contrast (Zeiss Axioscope 5; Zeiss Axiocam 208 color, Jena, Germany). Illustrations were made with the aid of a drawing tube (camera lucida) mounted on a Leica DM750 (Leica Microsystems, Wetzlar, Germany) microscope with phase contrast optics. Measurements (all in micrometers) are expressed as the mean followed by the range and the number of specimens analyzed in parentheses; body length includes the haptor. Measurements of the sclerotized structures (bars, anchors, accessory anchor sclerite, hooks, and copulatory complex) were performed according to Mizelle & Klucka [22] and the scheme shown in Figure 1. The copulatory complexes, comprising a sclerotized accessory piece and a male copulatory organ (MCO), and the direction of the MCO’s rings (counterclockwise vs. clockwise) followed Kritsky et al. [23]; the specific terminology of the genus *Tereancistrum* followed Kritsky et al. [6]; and the numbering and distribution of hook pairs followed Mizelle & Price [24]. Parameters of infestation of the parasites (prevalence and mean intensity of infestation) followed Bush et al. [25].

Type specimens, vouchers, and syngenophore [26] were deposited in the Helminthological Collection of the Instituto Oswaldo Cruz (CHIOC), state of Rio de Janeiro, Brazil, and in the Helminthological Collection of the Institute of Biosciences (CHIBB), of São Paulo State University—UNESP, in the municipality of Botucatu, state of São Paulo, Brazil. The following paratypes and vouchers were examined for comparative purposes; *Tereancistrum curimba* (CHIOC 36225, 36226, 36227a—b); *T. paranaensis* (CHIOC 37866 and 37867), and *T. pirassununguensis* (CHIOC 37816b—j).

### 2.3. Extraction, Amplification, and Sequencing of DNA

Total genomic DNA was extracted from the specimens individually, using the DNeasy Blood & Tissue Kit (Qiagen, Hilden, Germany), following the manufacturer’s recommended protocol. Fragments of the LSU rDNA gene were obtained through PCR amplifications using 10 μL of 2× MyFi^TM^ Mix (Bioline, Taunton, MA, USA), 1.5 µL of extracted DNA, 6.1 μL of pure water, and 1.2 µL of each PCR primer in a total volume of 20 μL. For amplification and sequencing, the following sets of primer pairs were used: U178 (5′-GCACCCGCTGAAYTTAAG-3′) and L1642 (5′-CCAGCGCCATCCATTTTCA-3′) [27], under the following cycling parameters: initial denaturation at 95 °C for 5 min, followed by 30 cycles at 95 °C for 30 s, annealing at 56 °C for 30 s, and extension at 72 °C for 2 min, followed by incubation at 72 °C for 10 min; and 382F (5′-AGCTGGTGGAGTCAAGCTTC3′) and 1289R (5′-TGCTCACGTTTGACGATCGA-3′) [28], under the following cycling parameters: initial denaturation at 95 °C for 5 min, followed by 30 cycles at 95 °C for 30 s, annealing at 47 °C for 30 s, and extension at 72 °C for 2 min, followed by incubation at 72 °C for 10 min. PCR products (2.0 μL) were run on an agarose gel (1%) using GelRed™ fluorescent nucleic acid dye added to BlueJuice^TM^ Gel Loading Buffer to confirm amplicon size and yield. PCR amplicons were purified using Agencourt AMPure XP magnetic beads (Beckman Coulter, Brea, CA, USA), following the manufacturer’s instructions. Automated sequencing was performed directly on purified PCR products using a BigDye v.3.1 Terminator Cycle Sequencing Ready Reaction kit on an ABI3730xl Genetic Analyzer (Applied Biosystems, Foster City, CA, USA). Contigs were assembled and edited in Sequencher v. 5.2.4 (Gene Codes, Ann Arbor, MI, USA) and then subjected to the BLAST algorithm to confirm identity.

### 2.4. Alignments and Phylogenetic Analyses

The newly obtained LSU rDNA sequences were aligned with other sequences from closely related dactylogyrid species retrieved from the GenBank database, plus sequences from species of the family Diplectanidae Monticelli, 1903, used as the outgroup (Appendix A). The alignment was constructed using the MUSCLE algorithm implemented using the software Geneious 7.1.3 [29] with default settings. The best model of nucleotide substitution for the dataset was estimated with the Akaike information criterion (AIC) implemented in jModelTest [30] as GTR + I + G. The phylogenetic analyses were performed using Bayesian inference (BI) and maximum likelihood (ML) methods. BI analysis was performed using MrBayes 3.2 [31] at the online interface Cyberinfrastructure for Phylogenetic Research (CIPRES) Science Gateway v3.3 [32]. The Markov chain Monte Carlo (MCMC) was run with two simultaneous runs for 10^6^ generations, sampling one tree every 100 generations, and with a “burn-in” Set to the first 25% of the trees. Nodes with posterior probability (pp) greater than 90 were considered well supported. The ML analysis was run in RAxML [33] at CIPRES Science Gateway v3.3 [32] with 1000 bootstrap replicates to support nodes. Nodes with bootstrap values (bv) above 70 were considered well supported. The BI and ML trees were visualized using FigTree v. 1.3.1 software [34] and edited using CorelDRAW X6. The genetic divergences among taxa were estimated in MEGA11 [35] using the Kimura 2-parameter model [36]. Rate variation among sites was modeled with a gamma distribution (shape parameter = 1). All ambiguous positions were removed for each sequence pair (pairwise deletion option).

## 3. Results

In the present study, nine species of *Tereancistrum* were found parasitizing the gills of fishes from the Salgado River sub-basin, state of Ceará, Brazil; of these, four new species are reported and described below. Furthermore, we provide information on the phylogenetic position among seven *Tereancistrum* species using phylogenetic analyses based on molecular data (LSU rDNA).

### 3.1. Amended Diagnosis

Class Monopisthocotyla Brabec, Salomaki, Scholz & Kuchta, 2023
Order Dactylogyridea Bychowsky, 1937
Family Dactylogyridae Bychowsky, 1933
*Tereancistrum* Kritsty, Thatcher & Kayton, 1980

Body divisible into cephalic region, trunk, peduncle, and haptor. Tegument thin and smooth. Cephalic area with lobes. Head organs, cephalic glands present. One or two pairs of eyes. Accessory granules absent or present at the level of the cephalic region. Pharynx muscular, glandular; esophagus bifurcating into two intestinal caeca; confluent posterior to gonads. Gonads intercaecal, tandem, or slightly overlapping; testis dorsal or posterior to ovary; seminal vesicle as dilation of vas deferens. Copulatory complex comprising articulated or nonarticulated male copulatory organ (MCO) and accessory piece; MCO tubular, sclerotized, either coiled or not. Counterclockwise or clockwise coiled MCO; accessory piece variable, serving as MCO guide distally. Ovary lying near mid-length; lateral (dextral or sinistral) vagina; seminal receptacle present; well-developed vitellaria. Haptor armed with 14 (7 pairs) hooks similar, dorsal and ventral pairs of anchors, with ancyrocephaline distribution [37]. Ventral anchors with accessory anchor sclerite articulated at the tip of the superficial root; accessory sclerite with a terminal spathulate portion. Ventral bar present, and dorsal bar present or absent. Parasitic on the gills of freshwater fishes.

Type species, host, and locality: *Tereancistrum kerri* Kritsky, Thatcher & Kayton, 1980 from *Brycon melanopterus* (Cope, 1872) (Characiformes: Bryconidae), Januacá Lake, state of Amazonas, Brazil.

*Remarks*: Kritsky, Thatcher & Kayton [6] proposed *Tereancistrum* to group dactylogyrids characterized by having spathulate accessory anchor sclerites associated with the ventral anchors. According to Kritsky et al. [6], all species allocated to this genus had a complete haptor formed by fourteen similar hooks, two bars (one ventral and one dorsal), and two pairs of anchors (one ventral and one dorsal). To date, this genus comprises 11 species that do not present a considerable diversity of structures. However, in the new species *Tereancistrum ancistrum* n. sp. described below, the dorsal bar is absent, thus justifying the amendment of this characteristic in the diagnosis of the genus.

### 3.2. Description of New Tereancistrum Species

**Figure 2:**
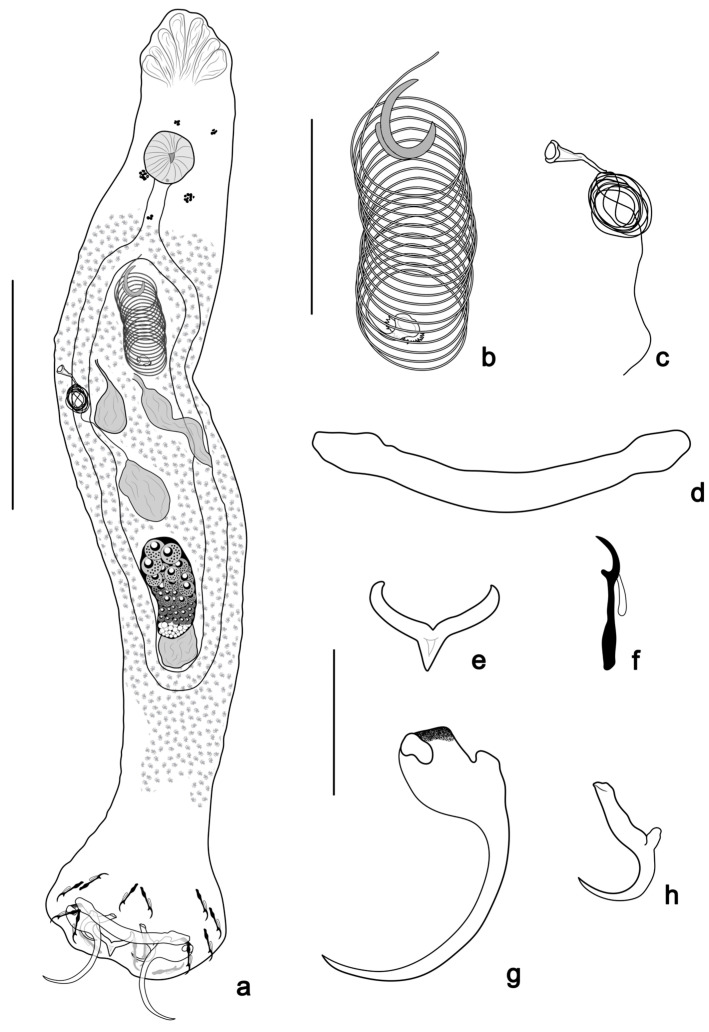
*Tereancistrum spiralocirrum* n. sp. (**a**) Whole mount in ventral view (composite); (**b**) copulatory complex (ventral view); (**c**) vagina; (**d**) ventral bar; (**e**) dorsal bar; (**f**) hooks; (**g**) ventral anchor with an accessory anchor sclerite; (**h**) dorsal anchor. Scale bars: (**a**) = 100 µm; (**b**,**c**) = 40 µm; (**d**–**h**) = 20 µm.

***Tereancistrum spiralocirrum* n. sp. Yamada, Sousa, Diniz & Yamada**

(Figure 2 and Figure 6a,b)

(urn:lsid:zoobank.org:act:9669F07B-8A50-4D03-A324-0543281581E0)

Type host: *Prochilodus brevis* Steindachner, 1875 (Characiformes: Prochilodontidae)

Type locality: Rosário weir, Salgado River basin, municipality of Lavras da Mangabeira, state of Ceará, Brazil (6°53′25.97″ S, 39°4′54.02″ W).

Other locality: Ubaldinho weir, Salgado River basin, municipality of Cedro, Ceará state, Brazil (6°35′5.58″ S, 39°14′22.27″ W).

Infestation site: Gills.

Infestation rate: Rosário weir: total number of hosts: 2; prevalence: 50%; total number of parasites: 2. Ubaldinho weir: total number of hosts: 14; prevalence: 35.71%; total number of parasites: 8; mean intensity: 1.6 ± 1.01; range of intensity: 1–3.

Specimens deposited: Holotype CHIOC 40616a and paratypes CHIOC 40616b, 40617–40620; and CHIBB 886L–888L.

Etymology: The specific name corresponds to the morphology of the MCO.

Description: (based on ten specimens mounted in Gray & Wess) body elongate, 486 (405–624; *n* = 5) long; greatest width 84 (70–90; *n* = 5), near mid-length. Cephalic lobes poorly developed; four pairs of head organs; cephalic glands not observed. Two pairs of eyes, posterior pair larger than anterior pair; few accessory granules dispersed in the cephalic region and anterior body. Pharynx subspherical, muscular, 24 (20–30; *n* = 8) in diameter; a long esophagus bifurcating into two intestinal caeca, confluent posterior to gonads. Gonads intercaecal, slightly overlapping. Ovary elongate, 58 (48–64; *n* = 6) long, 24 (16–33; *n* = 6) wide. Testis 33 (28–46; *n* = 6) long, 24 (15–33; *n* = 6) wide, dorsal to ovary. Prostatic reservoir ovate; seminal vesicle usually large, lying in midline immediately posterior to copulatory complex. Copulatory complex comprising a male copulatory organ (MCO) and an accessory piece. MCO coiled tube of 16 to 18 clockwise rings; diameter of first ring 20 (13–23; *n* = 10). Accessory piece 13 (12–16; *n* = 10) long, non-articulated with MCO base. Vagina dextral, comprising elongate, sclerotized, fine tube. Vagina aperture bugle-shaped. Seminal receptacle ovate. Peduncle elongate, haptor rectangular 53 (30–61; *n* = 8) long, 73 (61–80; *n* = 8) wide. Ventral anchor 34 (31–37; *n* = 10) long, 11 (8–14; *n* = 10) at base, with superficial roots robust, poorly developed deep root, evenly curved shaft and point. Accessory anchor sclerite short, 8 (5–10; *n* = 8) long, articulated with the superficial root of the ventral anchor. Dorsal anchor 15 (14–17; *n* = 10) long, 5 (4–6; *n* = 10) at base, with well-developed and elongate superficial root, short deep root, evenly curved shaft and point. Ventral bar 54 (50–57; *n* = 10) long, with slightly expanded ends. Dorsal bar Y-shaped, 17 (13–20; *n* = 9) long, 5 (4–6; *n* = 8) wide, with short posteromedian projection. Seven pairs of similar hooks, 18 (15–20; *n* = 56) long, with erect thumb, slightly curved shaft and point, inflated proximal portion of shank, filamentous hook (FH) loop approximately 1/3 shank length. Vitellaria coextensive with intestinal caeca, absent near reproductive organs. Oviduct, ootype, uterus, and eggs not observed.

Remarks: *Tereancistrum spiralocirrum* n. sp. was allocated to the genus *Tereancistrum* because it has an accessory anchor sclerite articulated with the ventral anchors. The new species resembles *T. pirassununguensis*, as it possesses a small accessory anchor sclerite recurved at the base of the ventral anchor and a Y-shaped dorsal bar. However, *Tereancistrum spiralocirrum* n. sp. can be easily distinguished from all congeners by the morphology of the MCO (a coiled tube with 16 to 18 clockwise rings).

**Figure 3:**
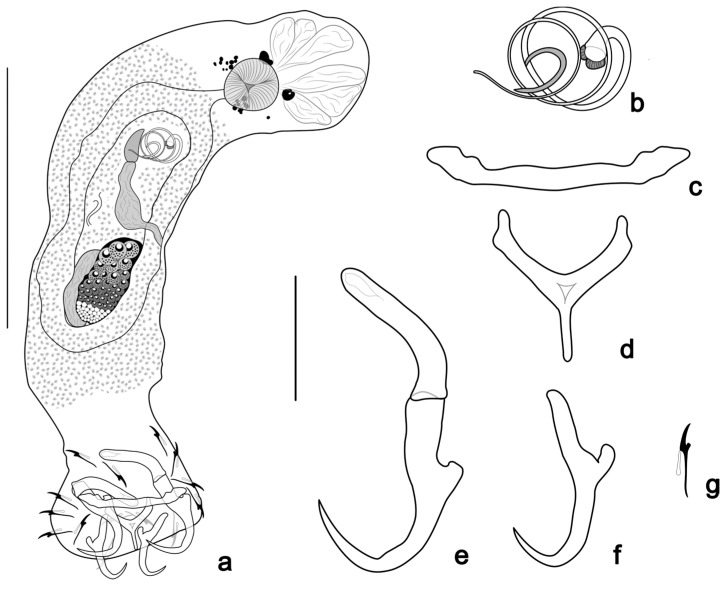
*Tereancistrum scleritelongatum* n. sp. (**a**) Whole mount in ventral view (composite); (**b**) copulatory complex (ventral view); (**c**) ventral bar; (**d**) dorsal bar; (**e**) ventral anchor with an accessory anchor sclerite; (**f**) dorsal anchor. (**g**) hooks. Scale bars: (**a**) = 100 µm; (**b**–**g**) = 20 µm.

***Tereancistrum scleritelongatum* n. sp. Yamada, Sousa, Diniz & Yamada**

(Figure 3 and Figure 6c)

(urn:lsid:zoobank.org:act:60470471-0F9B-46E6-9CD1-3C85D0E54C46)

Type host: *Prochilodus brevis* Steindachner, 1875 (Characiformes: Prochilodontidae)

Type locality: Ubaldinho weir, Salgado River basin, municipality of Cedro, state of Ceará, Brazil (6°35′5.58″ S, 39°14′22.27″ W).

Other locality: Lima Campos weir, Salgado River basin, municipality of Icó, state of Ceará, Brazil (6°22′59.99″ S, 38°58′0.01″ W); Rosário weir, Salgado River basin, municipality of Lavras da Mangabeira, state of Ceará, Brazil (6°53′25.97″ S, 39°4′54.02″ W).

Infestation site: Gills.

Infestation rate: Ubaldinho weir: total number of hosts: 14; prevalence: 50%; total number of parasites:11; mean intensity: 1.57 ± 0.93; range of intensity: 1–3. Lima Campos weir: total number of hosts: 25; prevalence: 12%; total number of parasites: 12; mean intensity: 6 ± 1.41; range of intensity: 5–7. Rosário weir: total number of hosts: 2; prevalence: 50%; total number of parasites: 1; range of intensity: 1.

Specimens deposited: Holotype CHIOC 40621 and paratypes CHIOC 40622–40626; and CHIBB 889L–891L.

Etymology: The specific name “*scleritelongatum*” refers to the large size of the accessory anchor sclerite articulated at the tip of the superficial root of the ventral anchor.

Description: (based on 17 specimens mounted in Gray & Wess) body elongate, 259 (204–305; *n* = 9) long; greatest width 63 (45–81; *n* = 9), near mid-length. Tegument smooth. Cephalic lobes poorly developed; three pairs of head organs; cephalic glands not observed.

Two pairs of eyes, one or both members of posterior pair sometimes lacking, component granules spherical, accessory granules dispersed in the cephalic region. Pharynx subspherical, muscular, 15 (12–17; *n* = 8) in diameter; short esophagus bifurcating into two intestinal caeca, confluent posterior to gonads. Gonads intercaecal, overlapping. Ovary elongate, 55 (42–65; *n* = 6) long, 21 (17–29; *n* = 6) wide. Testis 44 (38–48; *n* = 4) long, 23 (16–28; *n* = 4) wide, dorsal to ovary. Prostatic reservoir ovate. Copulatory complex comprising a MCO and free accessory piece. MCO coiled tube with 2½ to 3 clockwise rings; diameter of complete ring 16 (10–18; *n* = 17). Accessory piece 7 (5–10; *n* = 15) long, C-shaped, non-articulated with MCO base. Vagina dextral. Peduncle elongate, haptor sub-square 51 (40–66; *n* = 10) long, 55 (46–72; *n* = 10) wide. Ventral anchor 29 (27–31; *n* = 15) long, 7 (6–10; *n* = 15) at base, with superficial roots elongate and robust, poorly developed deep root, curved shaft and point. Accessory anchor sclerite well elongated, 25 (22–29; *n* = 15) long, articulated to superficial root of the ventral anchor, with spatulate end. Dorsal anchor 27 (26–29; *n* = 16) long, 5 (4–7; *n* = 16) at base, with well-developed superficial root, short deep root, evenly curved shaft and point. Ventral bar 41 (36–51; *n* = 15) long, straight with ends directed anteriorly. Dorsal bar Y-shaped, with posteromedian projection; 21 (16–33; *n* = 16) long, 13 (12–15; *n* = 13) wide. Seven pairs of similar hooks 13 (12–15; *n* = 34) long, with erect thumb, slightly curved point, straight shank, and FH loop half of the shank length. Vitellaria coextensive with intestinal caeca, absent near reproductive organs. Oviduct, ootype, uterus, seminal receptacle, vas deferens, and eggs not observed.

Remarks: *Tereancistrum scleritelongatum* n. sp. resembles the species *T. kerri* by having a dextral vagina, a ventral anchor with elongated and robust superficial roots, and an elongate, robust accessory anchor sclerite. However, the new species can be distinguished from *T. kerri* by having a Y-shaped dorsal bar, MCO with 2½ clockwise rings, and a free accessory piece (recurved dorsal bar; MCO with a simple tube and accessory piece articulated with MCO base in *T. kerri*).

**Figure 4:**
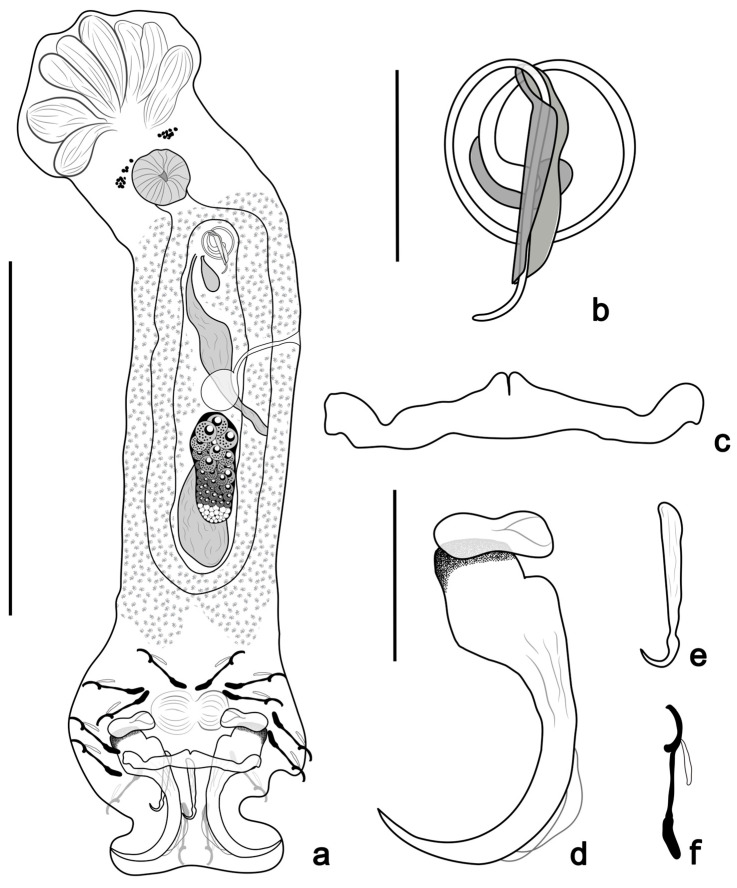
*Tereancistrum ancistrum* n. sp. (**a**) Whole mount in ventral view (composite); (**b**) copulatory complex (ventral view); (**c**) ventral bar; (**d**) ventral anchor with an accessory anchor sclerite; (**e**) dorsal anchor; (**f**) hooks. Scale bars: (**a**) = 100 µm; (**b**) = 10 µm; (**c**–**f**) = 20 µm.

***Tereancistrum ancistrum* n. sp. Yamada, Sousa, Diniz & Yamada**

(Figure 4 and Figure 6d)

(urn:lsid:zoobank.org:act:FFF7CD99-01C1-4D65-9A3D-A6FF7149890E)

Type host: *Prochilodus brevis* Steindachner, 1875 (Characiformes: Prochilodontidae)

Type locality: Lima Campos weir, Salgado River basin, municipality of Icó, state of Ceará, Brazil (6°22′59.99″ S, 38°58′0.01″ W).

Other locality: Ubaldinho weir, Salgado River basin, municipality of Cedro, Ceará state, Brazil (6°35′5.58″ S, 39°14′22.27″ W); Rosário weir, Salgado River basin, municipality of Lavras da Mangabeira, state of Ceará, Brazil (6°53′25.97″ S, 39°4′54.02″ W).

Infestation site: Gills.

Infestation rate: Lima Campos weir: total number of hosts: 25; prevalence: 72%; total number of parasites: 84; mean intensity: 4.67 ± 6.74; range of intensity: 1–29. Ubaldinho weir: total number of hosts: 14; prevalence: 78.57%; total number of parasites: 35; mean intensity: 3.18 ± 1.61; range of intensity: 1–9. Rosário weir: total number of hosts: 2; prevalence: 100%; total number of parasites: 4; mean intensity: 2.0 ± 1.4; range of intensity: 1–3.

Specimens deposited: Holotype CHIOC 40627; paratypes CHIOC 40628a, 40629a–b, 40630a–b, 40631–40633; and CHIBB 892L–899L; syngenophore CHIOC 40628b.

Molecular data: LSU rDNA sequence obtained from one specimen (GenBank accession number PV166050).

Etymology: The specific name is from Greek (*ancistrum* = hook) and refers to the shape of the dorsal anchor.

Description: (based on 15 specimens mounted in Gray & Wess) body 272 (203–355; *n* = 11) long, slender, fusiform; greatest width 52 (36–64; *n* = 13), near mid-length. Two cephalic lobes well-developed; three pairs of bilateral head organs; cephalic glands not observed. Two eyespots; a few accessory granules dispersed in cephalic region. Pharynx subspherical, 15 (11–18; *n* = 10) in diameter; esophagus short. Gonads intercaecal, slightly overlapping. Ovary elongate, 52 (35–68; *n* = 5) long, 31 (22–40; *n* = 3) wide. Testis dorsal to ovary, 49 (41–58; *n* = 3) long, 20 (11–29; *n* = 5) wide. Prostatic reservoir ovate. Copulatory complex comprising an MCO and accessory piece non-articulated with MCO base. Male copulatory organ coiled tube of 1 (one) clockwise ring; ring diameter 9 (8–12; *n* = 13). Accessory piece 12 (10–16; *n* = 13) long, serving as guide for distal portion of male copulatory organ, with slight proximal expansion. Vagina sinistral slightly sclerotized; spherical seminal receptacle. Peduncle short, haptor variable 49 (33–77; *n* = 8) long, 62 (57–68; *n* = 8) wide. Ventral anchor 38 (35–39; *n* = 15) long, 12 (10–14; *n* = 15) at base, with robust superficial roots, inconspicuous deep root, evenly curved shaft and point. Accessory anchor sclerite, 14 (12–18; *n* = 15) long, robust, articulated to superficial root of the ventral anchor. Two haptoral muscles attached to accessory sclerites. Dorsal anchor 18 (16–22; *n* = 15) long, 2 (1–3; *n* = 15) at base, with inconspicuous superficial and deep root, straight base and shaft, well-curved point. Ventral bar 42 (38–47; *n* = 15) long, with anteromedian indentation and ends directed anteriorly. Dorsal bar absent. Seven pairs of hooks 18 (16–20; *n* = 28) long, similar in size and shape, with robust thumb, recurved point, inflated proximal portion of shank. FH loop approximately ⅓ shank length. Vitellaria coextensive with intestinal caeca, absent near reproductive organs. Oviduct, ootype, uterus, and eggs not observed.

Remarks: *Tereancistrum ancistrum* n. sp. can be easily distinguished from its congeners due to the morphology of its haptoral sclerotized structures: the dorsal anchor (with inconspicuous superficial and deep roots, straight base and shaft) and absence of the dorsal bar (present in all other species).

**Figure 5:**
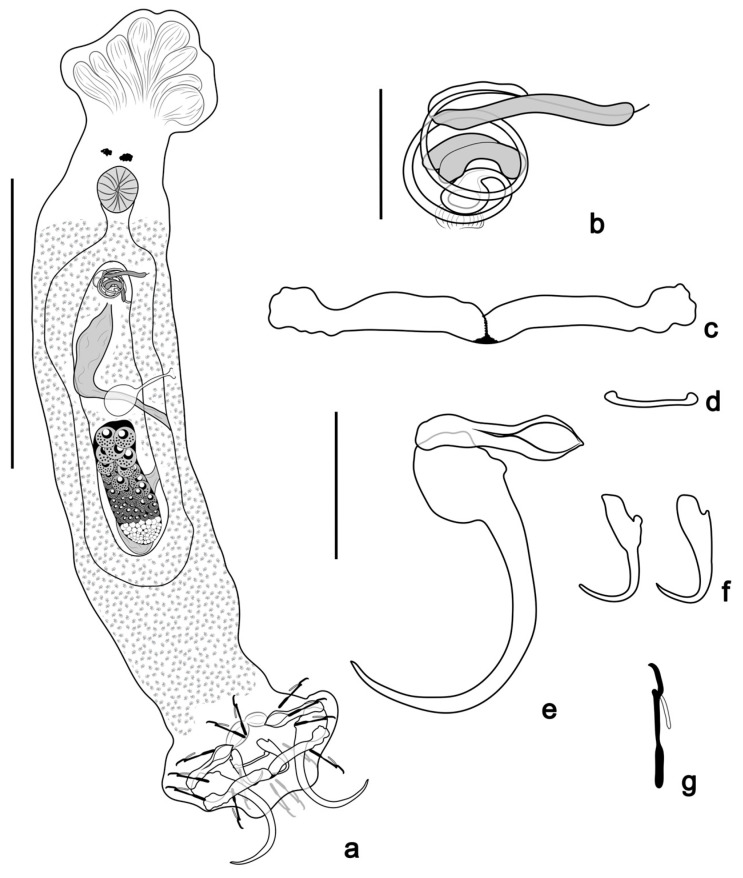
*Tereancistrum kritskyi* n. sp. (**a**) Whole mount in ventral view (composite); (**b**) copulatory complex (ventral view); (**c**) ventral bar; (**d**) dorsal bar; (**e**) ventral anchor with an accessory anchor sclerite; (**f**) dorsal anchor; (**g**) hooks. Scale bars: (**a**) = 100 µm; (**b**) = 10 µm; (**c**–**g**) = 20 µm.

**Figure 6:**
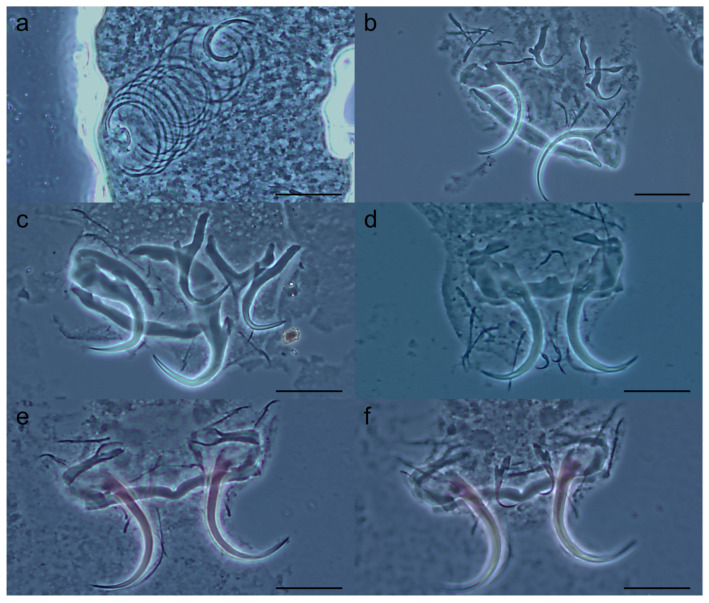
Photomicrographs of the sclerotized pieces of the four new species of *Tereancistrum* from the gills of *Prochilodus brevis* Steindachner, 1875 (Characiformes: Prochilodontidae) from the Salgado River basin, Caatinga domain, Brazil. *Tereancistrum spiralocirrum* n. sp.: (**a**) copulatory complex and (**b**) haptor. *Tereancistrum scleritelongatum* n. sp.: (**c**) haptor. *Tereancistrum ancistrum* n. sp.: (**d**) haptor. *Tereancistrum kritskyi* n. sp. (**e**) haptor (ventral view), and (**f**) haptor (dorsal view). Scale bars: (**a**–**f**) = 20 µm.

***Tereancistrum kritskyi* n. sp. Yamada, Sousa, Diniz & Yamada**

(Figure 5 and Figure 6e,f)

(urn:lsid:zoobank.org:act:6FE9047C-6484-475D-A9C6-C70FC3EFBC43)

Type host: *Prochilodus brevis* Steindachner, 1875 (Characiformes: Prochilodontidae).

Type locality: Lima Campos weir, Salgado River basin, municipality of Icó, state of Ceará, Brazil (6°22′59.99″ S, 38°58′0.01″ W).

Other locality: Ubaldinho weir, Salgado River basin, municipality of Cedro, Ceará state, Brazil (6°35′5.58″ S, 39°14′22.27″ W); Rosário weir, Salgado River basin, municipality of Lavras da Mangabeira, state of Ceará, Brazil (6°53′25.97″ S, 39°4′54.02″ W).

Infestation site: Gills.

Infestation rate: Lima Campos weir: total number of hosts: 24; prevalence: 48%; total number of parasites: 23; mean intensity: 1.92 ± 1.16; range of intensity: 1–4. Ubaldinho weir: total number of hosts: 14; prevalence: 78.57%; total number of parasites: 33; mean intensity: 3.0 ± 1.61; range of intensity: 1–6. Rosário weir: total number of hosts: 2; prevalence: 100%; total number of parasites: 8; mean intensity: 4.0 ± 2.83; range of intensity: 2–6.

Specimens deposited: Holotype CHIOC 40634; paratypes CHIOC 40635–40639; and CHIBB 900L–902L; syngenophore CHIOC 40640.

Molecular data: LSU rDNA sequence obtained from one specimen (GenBank accession number PQ481573).

Etymology: The specific epithet *kritskyi* is in honor of Dr. Delane C. Kritsky, Idaho State University, Pocatello, Idaho, USA, in recognition of his significant contributions to fish parasitology studies and as the descriptor of the genus *Tereancistrum*.

Description: (based on 12 specimens mounted in Gray & Wess) body elongate, 297 (252–350; *n* = 10) long; 54 (32–76; *n* = 9) near mid-length. Tegument smooth. Cephalic region well-developed, with two bilateral cephalic lobes; four pairs of head organs; cephalic glands not observed. Two eyespots, granules ovate, few accessory granules dispersed in cephalic region. Pharynx subspherical, muscular, 14 (11–19; *n* = 9) in diameter; short esophagus bifurcating into two intestinal caeca, confluent posterior to gonads. Gonads intercaecal, overlapping. Ovary elongate, 40 (31–46; *n* = 5) long, 15 (12–22; *n=* 5) wide. Testis dorsal to ovary 35 (27–44; *n* = 4) long, 18 (14–24; *n* = 5) wide; seminal vesicle usually large distal expansion of vas deferens, posterior to copulatory complex. Copulatory complex comprising MCO and free accessory piece. MCO coiled tube of 1½ clockwise rings; ring diameter 10 (8–14; *n* = 12). Accessory piece 8 (5–13; *n* = 11) long, non-articulated with MCO base. Vagina sinistral; seminal receptacle ovate. Peduncle elongate, haptor 51 (43–677; *n* = 10) long, 68 (56–82; *n* = 10) wide. Ventral anchor 38 (35–39; *n* = 12) long, 12 (9–14; *n* = 12) base, inconspicuous superficial roots, absent deep root, curved shaft and point. Accessory anchor sclerite 21 (17–23; *n* = 12) long, spatula-shape, articulated to superficial root of the ventral anchor. Two haptoral muscles attached to accessory sclerites. Dorsal anchor 14 (13–15; *n* = 12) long, 3 (2–4; *n* = 12) at base, with elongate superficial root, short deep root, straight shaft, and curved point. Ventral bar 55 (49–60; *n* = 11) long, straight with ends directed anteriorly. Dorsal bar 13 (10–16; *n* = 8) long. Seven pairs of hooks 17 (15–20; *n* = 29) long, similar in size and shape, with erect thumb, slightly curved shaft and point, inflated proximal portion of shank, FH loop approximately ¼ shank length. Vitellaria coextensive with intestinal caeca, absent near reproductive organs. Oviduct, ootype, uterus, prostatic reservoir, and eggs not observed.

Remarks: *Tereancistrum kritskyi* n. sp. closely resembles all congeners by presenting an accessory anchor sclerite articulated to the superficial root of the ventral anchor. The new species resembles *T. paranaensis* by having a sinistral vagina and by the morphology of haptoral structures (ventral bar elongate, dorsal bar straight with slightly expanded ends, and dorsal anchor with elongated superficial root). However, the new species differs from *T. paranaensis* by possessing an MCO with a coiled tube of 1½ clockwise rings, having a delicate dorsal anchor measuring 14 (13–15; *n* = 12) long and 3 (2–4; *n* = 12) wide, and it parasitizes the gills of a prochilodontid; *T. paranaensis* has an MCO with 2½ clockwise rings and a robust dorsal anchor measuring 24 (20–28; *n* = 10) long and 7 (6–8; *n* = 10) wide, and it parasitizes the gills of an anostomid [10,38]. The examination of paratypes of *T. paranaensis* described by Karling et al. [10] confirms the resemblances and differences reported in the current study.

### 3.3. Previously Known Tereancistrum Species Used in Molecular Analysis

***Tereancistrum curimba* Lizama, Takemoto & Pavanelli, 2004**

(Figure 7a,b)

Host: *Prochilodus brevis* Steindachner, 1875 (Characiformes: Prochilodontidae).

Localities: Lima Campos weir, Salgado River basin, municipality of Icó, state of Ceará, Brazil (6°22′59.99″ S, 38°58′0.01″ W); Ubaldinho weir, Salgado River basin, municipality of Cedro, state of Ceará, Brazil (6°35′5.58″ S, 39°14′22.27″ W); Rosário weir, Salgado River basin, municipality of Lavras da Mangabeira, state of Ceará, Brazil (6°53′25.97″ S, 39°4′54.02″ W).

Infestation site: Gills.

Infestation rate: Lima Campos weir: total number of hosts: 24; prevalence: 68%; total number of parasites: 69; mean intensity: 4.06 ± 4.04; range of intensity: 1–15. Ubaldinho weir: total number of hosts: 14; prevalence: 92.86%; total number of parasites: 67; mean intensity: 5.15 ± 3.85; range of intensity: 1–12. Rosário weir: total number of hosts: 2; prevalence: 100%; total number of parasites: 10; mean intensity: 5; range of intensity: 5.

Specimens deposited: Syngenophore CHIOC 40642; vouchers CHIOC 40641a–c, 40643; and CHIBB 903L–905L.

Molecular data: LSU rDNA sequences were obtained from two specimens collected from Lima Campos weir (GenBank accession numbers: PQ481571 and PQ481572).

***Tereancistrum pirassununguensis* Cepeda, Ceccarelli & Luque, 2012**

(Figure 7c,d)

Host: *Prochilodus brevis* Steindachner, 1875 (Characiformes: Prochilodontidae).

Localities: Lima Campos weir, Salgado River basin, municipality of Icó, state of Ceará, Brazil (6°22′59.99″ S, 38°58′0.01″ W); Ubaldinho weir, Salgado River basin, municipality of Cedro, state of Ceará, Brazil (6°35′5.58″ S, 39°14′22.27″ W); Rosário weir, Salgado River basin, municipality of Lavras da Mangabeira, state of Ceará, Brazil (6°53′25.97″ S, 39°4′54.02″ W).

Infestation site: Gills.

Infestation rate: Lima Campos weir: total number of hosts: 24; prevalence: 84%; total number of parasites: 263; mean intensity: 12.52 ± 4.75; range of intensity: 1–105. Ubaldinho weir: total number of hosts: 14; prevalence: 92.86%; total number of parasites: 105; mean intensity: 8.08 ± 2.17; range of intensity: 1–26. Rosário weir: total number of hosts: 2; prevalence: 100%; total number of parasites: 28; mean intensity: 14.0 ± 11.0; range of intensity: 3–25.

Specimens deposited: Syngenophore CHIOC 40645; vouchers CHIOC 40644, 40646a–b, 40647a–c, 40648–40650; and CHIBB 906L–909L.

Molecular data: LSU rDNA sequences were obtained from two specimens collected from Lima Campos weir (GenBank accession numbers PQ889561 and PQ889562).

***Tereancistrum takemotoi* Leite, Pelegrini, Azevedo & Abdallah, 2020**

(Figure 7e,f)

Host: *Prochilodus brevis* Steindachner, 1875 (Characiformes: Prochilodontidae).

Localities: Lima Campos weir, Salgado River basin, municipality of Icó, state of Ceará, Brazil (6°22′59.99″ S, 38°58′0.01″ W); Ubaldinho weir, Salgado River basin, municipality of Cedro, state of Ceará, Brazil (6°35′5.58″ S, 39°14′22.27″ W); Rosário weir, Salgado River basin, municipality of Lavras da Mangabeira, state of Ceará, Brazil (6°53′25.97″ S, 39°4′54.02″ W).

Infestation site: Gills.

Infestation rate: Lima Campos weir: total number of hosts: 24; prevalence: 76%; total number of parasites: 56; mean intensity: 2.95 ± 1.51; range of intensity: 1–6. Ubaldinho weir: total number of hosts: 14; prevalence: 100%; total number of parasites: 126; mean intensity: 9.0 ± 6.84; range of intensity: 2–23. Rosário weir: total number of hosts: 2; prevalence: 100%; total number of parasites: 5; mean intensity: 2.5 ± 0.71; range of intensity: 2–3.

Specimens deposited: Syngenophore CHIOC 40651a; vouchers CHIOC 40651b, 40652, 40653; and CHIBB 910L–913L.

Molecular data: LSU rDNA sequence obtained from one specimen collected from Lima Campos weir (GenBank accession number PQ481575).

***Tereancistrum paranaensis* Karling, Lopes, Takemoto & Pavanelli, 2014**

(Figure 7g,h)

Host: *Leporinus piau* Fowler, 1941 (Characiformes: Anostomidae).

Locality: Lima Campos weir, Salgado River basin, municipality of Icó, state of Ceará, Brazil (6°22′59.99″ S, 38°58′0.01″ W).

Infestation site: Gills.

Infestation rate: Lima Campos weir: total number of hosts: 35; prevalence: 88.57%; total number of parasites: 167; mean intensity: 5.39 ± 1.05; range of intensity: 1–31.

Specimens deposited: Syngenophore CHIOC 40654; vouchers CHIOC 40655a–b, 40656; and CHIBB 914L–916L.

Molecular data: LSU rDNA sequences were obtained from two specimens (GenBank accession numbers PQ889563 and PQ889564).

***Tereancistrum parvus* Kritsky, Thatcher & Kayton, 1980**

(Figure 7i,j)

Host: *Leporinus piau* Fowler, 1941 (Characiformes: Anostomidae).

Locality: Lima Campos weir, Salgado River basin, municipality of Icó, state of Ceará, Brazil (6°22′59.99″ S, 38°58′0.01″ W).

Infestation site: Gills.

Infestation rate: Lima Campos weir: total number of hosts: 35; prevalence: 88.57%; total number of parasites: 144; mean intensity: 4.65 ± 3.39; range of intensity: 1–15.

Specimens deposited: Syngenophore CHIOC 40657; vouchers CHIOC 40658–40660; and CHIBB 917L–919L.

Molecular data: LSU rDNA sequence was obtained from one specimen (GenBank accession number PQ481574).

**Figure 7:**
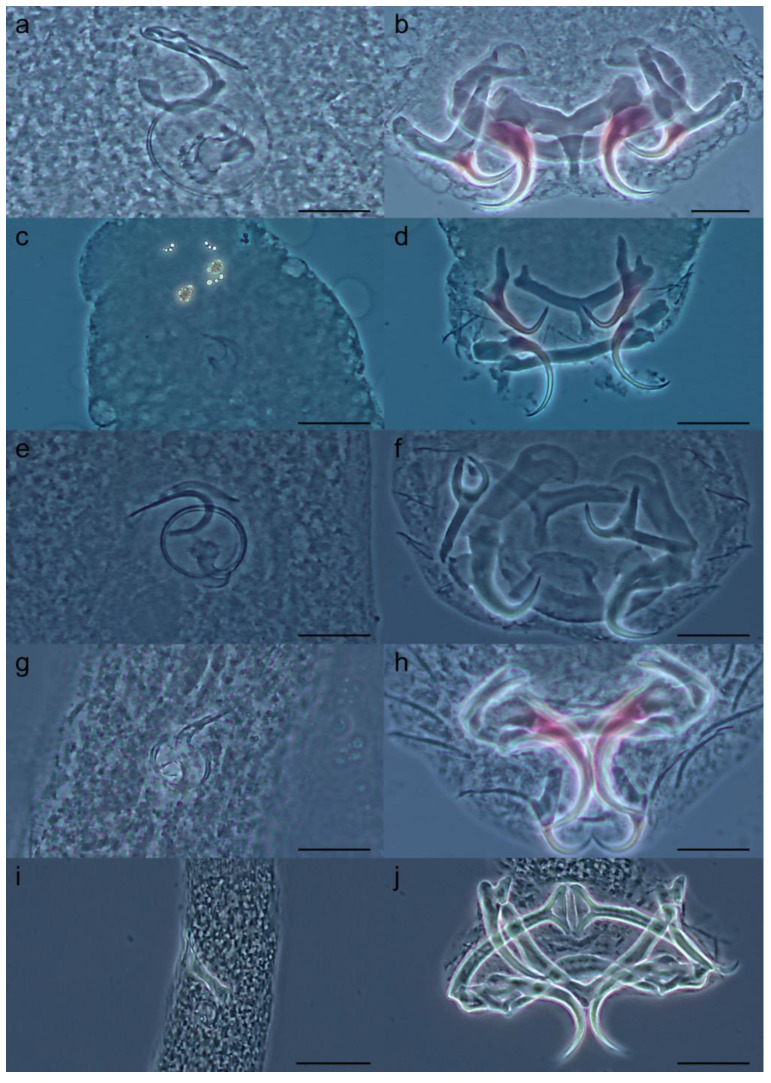
Photomicrographs of the sclerotized pieces of the *Tereancistrum* spp. from gills of the fishes from the Salgado River basin, Caatinga domain, Brazil. *Tereancistrum curimba*: (**a**) copulatory complex and (**b**) haptor. *Tereancistrum pirassununguensis*: (**c**) copulatory complex and (**d**) haptor. *Tereancistrum takemotoi*: (**e**) copulatory complex and (**f**) haptor. *Tereancistrum paranaensis*: (**g**) Copulatory complex and (**h**) haptor. *Tereancistrum parvus:* (**i**) copulatory complex and (**j**) haptor. Scale bars: (**a**–**h**) = 20 µm; (**i**,**j**) = 40 µm.

### 3.4. Molecular Data and Phylogenetic Inferences

**Figure 8:**
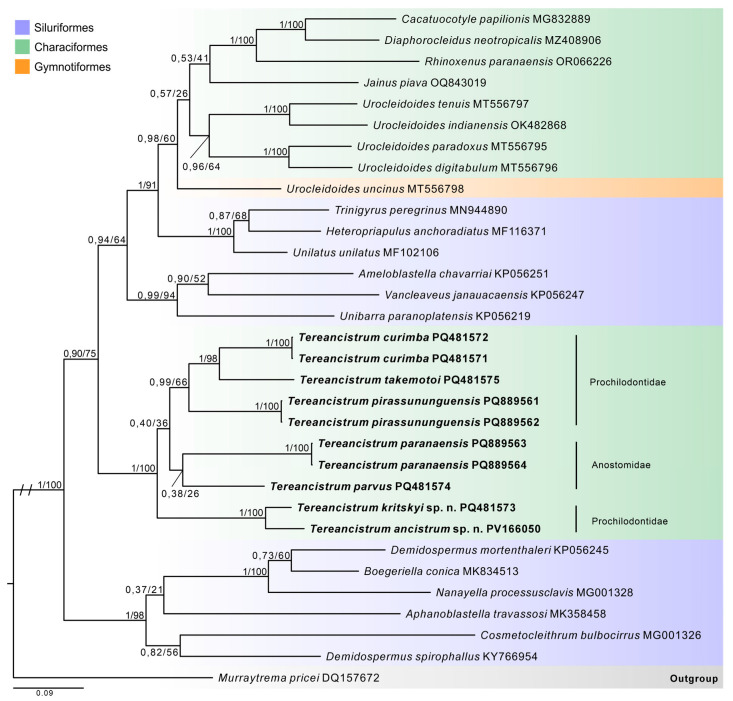
Bayesian inference phylogram based on partial sequences of LSU rDNA showing the position of newly sequenced *Tereancistrum* spp. (in bold) among selected dactylogyrids. GenBank accession numbers are given after species names. Support values are included before nodes as follows: posterior probabilities for Bayesian inference/bootstrap for the maximum likelihood analyses. Only nodes with posterior probability > 0.90 and bootstrap values > 70 are considered supported. Branch length scale bar indicates the number of substitutions per site.

Ten partial LSU rDNA sequences of *Tereancistrum* spp. were successfully obtained: two specimens of *T. curimba* (PQ481571, 1485 bp; PQ481572, 1468 bp), one of *T. takemotoi* (PQ481575, 1492 bp), two of *T. pirassununguensis* (PQ889561, 1510 bp; PQ889562, 1511 bp), one of *T. parvus* (PQ481574, 1438 bp), two of *T. paranaensis* (PQ889563, 1508 bp; PQ889564, 1485 bp), one of *Tereancistrum kritskyi* n. sp. (PQ481573, 1310 bp), and one of *Tereancistrum ancistrum* n. sp. (PV166050, 730 bp). Unfortunately, we were unable to obtain sequences from specimens of *Tereancistrum spiralocirrum* n. sp. and *Tereancistrum scleritelongatum* n. sp. The final alignment consisted of 32 sequences with 1175 nucleotides in total length. The LSU rDNA phylogenetic analyses inferred with BI and ML (Figure 8) showed that the ten new sequences of *Tereancistrum* spp. were resolved as monophyletic, with maximum support in both analyses (pp = 1; bv = 100); *Tereancistrum curimba* was placed as sister species of *T. takemotoi*, and *T. pirassununguensis* was recovered as the closest related to these two species, while *T. parvus* and *T. paranaensis* were recovered as closely related and sisters to the aforementioned clade.

The two new species *Tereancistrum kritskyi* n. sp. and *Tereancistrum ancistrum* n. sp. were grouped together as sister species and closely related to the other eight *Tereancistrum* spp. sequences. The *Tereancistrum* clade was recovered as the earliest divergent (pp = 90; bv = 75) of a clade containing the genera *Urocleidoides* Mizelle & Price, 1964, *Jainus* Mizelle, Kritsky & Crane, 1968, *Cacatuocotyle* Boeger, Domingues & Kritsky, 1997, *Diaphorocleidus* Jogunoori, Kritsky & Venkatanarasaiah, 2004, *Rhinoxenus* Kritsky, Thatcher & Boeger, 1988, *Trinigyrus* Hanek, Molnar & Fernando, 1974, *Heteropriapulus* Kritsky, 2007, *Unilatus* Mizelle & Kritsky, 1967, *Characithecium* Mendoza-Franco, Reina & Torchin, 2009, *Ameloblastella* Kritsky, Mendoza-Franco & Scholz, 2000, *Vancleaveus* Kritsky, Thatcher & Boeger, 1986 and *Unibarra* Suriano & Incorvaia, 1995—parasites of Characiformes, Siluriformes, and Gymnotiformes; another clade containing the sequences of genera *Cosmetocleithrum* Kritsky, Thatcher and Boeger, 1986, *Demidospermus* Suriano, 1983, *Aphanoblastella* Kritsky, Mendoza-Franco & Scholz, 2000, *Boegeriella* (Mendoza-Palmero, Mendoza-Franco, Acosta & Scholz, 2019), and *Nanayella* Acosta, Mendoza-Palmero, Silva & Scholz, 2019—all parasites of Siluriformes—was recovered as sister group of the aforementioned clade (pp = 1; bv = 100).

The intraspecific genetic divergence (Supplementary Material Table S1) among the sequences of *T. curimba*, *T. pirassununguensis*, and *T. paranaensis* was null, whereas the range of interspecific divergence between the sequences of *Tereancistrum* spp. varied from 8% (*Tereancistrum kritskyi* n. sp. versus *Tereancistrum ancistrum* n. sp.) to 32% (*T. pirassununguensis* versus *Tereancistrum ancistrum* n. sp.).

## 4. Discussion

According to Kritsky et al. [6], *Tereancistrum* is characterized by possessing: (1) gonads in tandem or slightly overlapping; (2) copulatory complex comprising a MCO and an accessory piece; (3) haptor armed with seven pairs of similar hooks; (4) two pairs of anchors with accessory anchor sclerites associated with the ventral anchors; (5) dorsal and ventral bars; and (6) one or two pairs of eyes. Among the four new species proposed in this study, *Tereancistrum spiralocirrum* n. sp. has the greatest number of rings in the MCO (with 16 to 18 rings), while *Tereancistrum ancistrum* n. sp., *T. curimba*, *T. flabellum*, *Tereancistrum kritskyi* n. sp., *T. ornatus*, *T. paranaensis*, *T. parvus*, *T. pirassununguensis*, *Tereancistrum scleritelongatum* n. sp., *T. takemotoi*, *T. toksonum*, and *T. campanum* have a coiled tube MCO comprising 1 to 3½ rings. *Tereancistrum kerri* and *T. ornatus* are the only two species that possess an accessory piece articulated with the MCO base. Furthermore, *Tereancistrum ancistrum* n. sp. is the only species of the *Tereancistrum* with the absence of a dorsal bar, which has never been verified in other congeners. Therefore, this additional characteristic observed in *Tereancistrum ancistrum* n. sp. required an amendment to the generic diagnosis of *Tereancistrum* to accommodate the new species proposed in the present study.

Considering all the valid species of *Tereancistrum*, the presence of an accessory anchor sclerite articulated to the superficial root of the ventral anchor is one of the main diagnostic characteristics of the genus [15]. The accessory anchor sclerite has a variable shape and size among the congeners: it can be reduced (*T. pirassununguensis* and *Tereancistrum spiralocirrum* n. sp.), well-elongated (*Tereancistrum scleritelongatum* n. sp., *T. flabellum*, *T. parvus*, and *T. campanum*), or robust (*T. kerri*, *T. toksonum*, *T. takemotoi*, *T. curimba*, and *Tereancistrum kritskyi* n. sp.). Also, *Tereancistrum ancistrum* n. sp., *Tereancistrum kritskyi* n. sp., *T. paranaensis*, *T. flabellum*, and *T. parvus* possess a pair of eyespots, while the other species present two pairs of eyespots.

Our phylogenetic analysis, including ten new sequences of the LSU rDNA gene obtained for *Tereancistrum* spp., along with published sequences of members of Dactylogyridae, supports the monophyly of *Tereancistrum*. The phylogenetic position of *Tereancistrum* within Dactylogyridae was first established by analysis of LSU rDNA sequences. *Tereancistrum ancistrum* n. sp. and *Tereancistrum kritskyi* n. sp., both parasites of *P. brevis* (Prochilodontidae), formed the earliest divergent clade and were recovered as sister species, showing the lowest genetic divergence (8%) among all *Tereancistrum* sequences analyzed. These species resemble each other in body size and shape, as well as in the morphology of the copulatory complex and internal organs; however, *Tereancistrum ancistrum* n. sp. is distinguished by the absence of a dorsal bar. The other three *Tereancistrum* species from *P. brevis* (*T. curimba*, *T. pirassununguensis*, and *T. takemotoi*) clustered in a separate clade, which was positioned as sister to *T. paranaensis* and *T. parvus*, parasites of *L. piau* (Anostomidae), though with low support. The most genetically divergent taxa were *T. ancistrum* n. sp. and *T. pirassununguensis*, which differ by 32% in the LSU rDNA gene despite parasitizing the same host species (*P. brevis*) and co-occurring in the same geographic locality. These two species share general morphological features, such as the copulatory complex and internal organs, but they differ markedly in the haptoral structures. The coexistence of five congeneric species of *Tereancistrum* on *P. brevis* in a single locality suggests a complex evolutionary history involving both sympatric and synxenic processes. The genetic divergences observed among species (especially high between *T. ancistrum* n. sp. and *T. pirassununguensis*) support the hypothesis of independent evolutionary origins. The phylogenetic clustering of *Tereancistrum* species associated with *P. brevis* and *L. piau* may further emphasize the role of host specificity in parasite diversification. Further investigations using more resolutive molecular markers such as COI mtDNA, combined with ecological data, may help clarify the mechanisms underlying the diversity and coevolutionary processes of these species.

Hasuike et al. [13] delivered the first phylogenetic analysis using sequences of the COI mtDNA gene of two *Tereancistrum* spp., i.e., *T. campanum* and *T. kerri*, collected from *Brycon nattereri* Günther, 1864 in the Tocantins-Araguaia River basin, located in the state of Goiás, Brazil. These two species presented a genetic distance ranging from 22.6% to 25.4%. In our study, all *Tereancistrum* specimens used for sequencing were collected from the prochilodontid *P. brevis* and the anostomid *L. piau* from the Brazilian Caatinga domain and sequenced only for the LSU rDNA gene; in future studies, we should gather more *Tereancistrum* spp. from a wider spectrum of host species and localities, including the type species *T. kerri*, along with new sequences from other molecular markers, such as the COI mtDNA gene, in order to compare our results to other previous studies and investigate coevolutionary and species delimitation processes.

This study describes the diversity of *Tereancistrum* spp. that parasitize the gills of prochilodontids and anostomids from the Caatinga domain in Brazil. Based on morphological and molecular characterization, we elevate the number of valid species currently comprised in the genus to 15 by erecting four new species of *Tereancistrum*: *Tereancistrum spiralocirrum* n. sp., *Tereancistrum scleritelongatum* n. sp., *Tereancistrum ancistrum* n. sp. and *Tereancistrum kritskyi* n. sp. (see Table 1). An additional morphological characteristic, i.e., the absence of a dorsal bar, observed in *Tereancistrum ancistrum* n. sp. required us to extend the generic diagnostic features and propose an amendment to the genus. Our molecular analyses, combined with morphological traits, were crucial to investigating and presenting the first phylogenetic position of *Tereancistrum* within Dactylogyridae based on LSU rDNA, contributing new information to a growing genetic library of Dactylogyridae, which will allow future studies to explore and evaluate the phylogenetic relationships among the genera within it.

## Figures and Tables

**Table 1:** List of *Tereancistrum* species, their hosts (Characiformes), and geographical distribution in the Neotropical region. The genus name is abbreviated after being mentioned for the first time.

Species	Host Species	Host Family	Locality	References
*Tereancistrum ancistrum* n. sp.	*Prochilodus brevis* Steindachner, 1875	Prochilodontidae	Lima Campos weir, Salgado River basin, Ceará, Brazil	Present study
*T. arcuatus* Cohen, Kohn & Boeger, 2012	*Salminus brasiliensis* (Cuvier, 1816)	Bryconidae	Paraná River, Upper Paraná River basin, Paraná, Brazil	[9]
*T. campanum* Hasuike, Scorsim, Arjona, Amaral, Damacena-Silva, Araújo, Bellay, Oliveira & Takemoto, 2025	*Brycon nattereri* Günther, 1864	Bryconidae	Traíras River, Tocantins-Araguaia River basin, Goiás, Brazil	[13]
*T. curimba* Lizama, Takemoto & Pavanelli, 2004	*P. lineatus* (Valenciennes, 1837)	Prochilodontidae	Paraná River, Upper Paraná River basin, Paraná, Brazil	[7]
*T. flabellum* Zago, Yamada, Franceschini, Bongiovani, Yamada & Silva, 2017	*Leporinus friderici* (Bloch, 1794)	Anostomidae	Sapucaí-Mirim River, Grande River basin, São Paulo, Brazil	[11]
*T. kerri* Kritsky, Thatcher & Kayton, 1980 (type species)	*B. melanopterus* (Cope, 1872)	Bryconidae	Januaca Lake, Amazonas, Brazil	[6]
*Tereancistrum kritskyi* n. sp.	*P. brevis*	Prochilodontidae	Lima Campos weir, Salgado River basin, Ceará, Brazil	Present study
*T. ornatus* Kritsky, Thatcher & Kayton, 1980	*P. reticulatus* Valenciennes, 1850	Prochilodontidae	Cauca River, Colombia Brazil	[6]
*T. paranaensis* Karling, Lopes, Takemoto & Pavanelli, 2014	*Schizodon borellii* (Boulenger, 1900)	Anostomidae	Paraná River, Upper Paraná River basin, Paraná, Brazil	[10]
*T. parvus* Kritsky, Thatcher & Kayton, 1980	*L. fasciatus* (Bloch, 1792)	Anostomidae	Amazon River Basin, Brazil	[6]
*T. pirassununguensis* Cepeda, Ceccarelli & Luque, 2012	*P. lineatus*	Prochilodontidae	Mogi Guaçu River, São Paulo, Brazil	[8]
*Tereancistrum scleritelongatum* n. sp.	*P. brevis*	Prochilodontidae	Ubaldinho weir, Salgado River basin, Ceará, Brazil	Present study
*Tereancistrum spiralocirrum* n. sp.	*P. brevis*	Prochilodontidae	Rosário weir, Salgado River basin, Ceará, Brazil	Present study
*T. takemotoi* Leite, Pelegrini, Azevedo & Abdallah, 2020	*P. lineatus*	Prochilodontidae	Batalha River, Tietê-Batalha River basin, São Paulo, Brazil	[12]
*T. toksonum* Lizama, Takemoto & Pavanelli, 2004	*P. lineatus*	Prochilodontidae	Paraná River, Upper Paraná River basin, Paraná, Brazil	[7]

## Data Availability

All data relating to this research are available in the article.

## Supplementary Materials

The following supporting information can be downloaded at: https://www.mdpi.com/article/10.3390/pathogens14050467/s1, Table S1: Estimates of Evolutionary Divergence between Sequences. References [35,36,39] are cited in the supplementary materials.

## Appendix A

**Table A1:** List of monopisthocotylans included in the phylogenetic analyses, with details of the host, locality, and GenBank accession numbers. New sequences obtained for the present study are in bold.

Monopisthocotylan Species	Host Species	Locality	LSU rDNA	References
Dactylogyridae				
*Ameloblastella chavarriai* (Price, 1938)	*Rhamdia quelen* (Quoy & Gaimard, 1824)	Mexico	KP056251	[14]
*Aphanoblastella travassosi* (Price, 1938)	*Rhamdia guatemalensis* (Günther, 1864)	Mexico	MK358458	[14]
*Boergeriella conica* Mendoza-Palmero, Mendoza-Franco, Acosta & Scholz, 2019	*Platynematichthys notatus* (Jardine, 1841)	Peru	MK834513	[40]
*Cacatuocotyle papilionis* Zago, Franceschini, Müller & Silva, 2018	*Astyanax lacustris* (Lütken, 1875)	Brazil	MG832889	[41]
*Cosmetocleithrum bulbocirrus* Kritsky, Thatcher& Boeger, 1986	*Pterodoras granulosus* (Valenciennes, 1821)	Brazil	MG001326	[42]
*Demidospermus mortenthaleri* Mendoza-Palmero, Scholz, Mendoza-Franco & Kuchta, 2012	*Brachyplatystoma juruense* (Boulenger, 1898)	Peru	KP056245	[14]
*Demidospermus spirophallus* Franceschini, Zago, Müller, Francisco, Takemoto & Silva, 2017	*Proloricaria prolixa* (Isbrücker & Nijssen, 1978)	Brazil	KY766954	[43]
*Diaphorocleidus neotropicalis* Zago et al. 2021	*A. lacustris*	Brazil	MZ408906	[44]
*Heteropriapulus anchoradiatus* Acosta, Franceschini, Zago, Scholz & Silva, 2017	*Pterygoplichthys ambrosettii* (Holmberg, 1893)	Brazil	MF116371	[45]
*Jainus piava* Karling, Bellay, Takemoto & Pavanelli, 2011	*Schizodon nasutus* Kner, 1858	Brazil	OQ843019	[28]
*Nanayella processusclavis* Acosta, Mendoza-Palmero, Silva & Scholz, 2019	*Hemisorubim platyrhynchos* (Valenciennes, 1840)	Brazil	MG001328	[42]
*Rhinoxenus paranaensis* Rossin & Timi, 2019	*Serrasalmus maculatus* Kner, 1858	Brazil	OR066226	[46]
*Trinigyrus peregrinus* Nitta & Nagasawa, 2016	*P. ambrosettii*	Brazil	MN944890	[47]
***Tereancistrum ancistrum* n. sp.**	***Prochilodus brevis* Steindachner, 1875**	**Brazil**	**PV166050**	**Present study**
***Tereancistrum curimba* Lizama, Takemoto & Pavanelli, 2004**	***P. brevis***	**Brazil**	**PQ481571-72**	**Present study**
***Tereancistrum kritskyi* n. sp.**	***P. brevis***	**Brazil**	**PQ481573**	**Present study**
***Tereancistrum parvus* Kritsky, Thatcher & Kayton, 1980**	***Leporinus piau* Fowler, 1941**	**Brazil**	**PQ481574**	**Present study**
***Tereancistrum takemotoi* Leite, Pelegrini, Azevedo & Abdallah, 2020**	***P. brevis***	**Brazil**	**PQ481575**	**Present study**
***Tereancistrum paranaensis* Karling, Lopes, Takemoto & Pavanelli, 2014**	***P. brevis***	**Brazil**	**PQ889563-64**	**Present study**
***Tereancistrum pirassununguensis* Cepeda, Ceccarelli & Luque, 2012**	***P. brevis***	**Brazil**	**PQ889561-62**	**Present study**
*Unibarra paranoplatensis* Suriano & Incorvaia, 1995	*Aguarunichthys torosus* Stewart, 1986	Peru	KP056219	[14]
*Unilatus unilatus* Mizelle & Kritsky, 1967	*P. ambrosettii*	Brazil	MF102106	[42]
*Urocleidoides digitabulum* Zago et al. 2020	*Megaleporinus elongatus* (Valenciennes, 1850)	Brazil	MT556796	[18]
*Urocleidoides indianensis* Oliveira, Silva, Vieira & Acosta, 2021	*Parodon nasus* Kner, 1859	Brazil	OK482868	[48]
*Urocleidoides paradoxus* Kritsky, Thatcher & Boeger, 1986	*Leporinus friderici* (Bloch, 1794)	Brazil	MT556795	[18]
*Urocleidoides tenuis* Zago et al. 2020	*Apareiodon piracicabae* (Eigenmann, 1907)	Brazil	MT556797	[18]
*Urocleidoides uncinus* Zago et al. 2020	*Gymnotus inaequilabiatus* (Valenciennes, 1839)	Brazil	MT556798	[18]
*Vancleaveus janauacaensis* Kritsky, Thatcher & Boeger, 1986	*P. granulosus*	Peru	KP056247	[14]
Diplectanidae				
*Murraytrema pricei* Bychowsky & Nagibina, 1977	*Nibea albiflora* Richardson, 1846	China	DQ157672	[49]

## References

[1] Froese R., Pauly D., FishBase World Wide Web Electronic Publication. https://www.fishbase.org.

[2] Albert J.S., Tagliacollo V.A., Dagosta F. (2020). Diversification of Neotropical Freshwater Fishes. Annu. Rev. Ecol. Evol. Syst..

[3] Buckup P.A., Menezes N.A., Ghazzi M.A.S. (2007). Catálogo das Espécies de Peixes de Água Doce do Brasil.

[4] Gimênes J.R.H., Rech R. (2022). Guia Ilustrado dos Peixes do Pantanal e Entorno.

[5] Cohen S.C., Justo M.C.N., Kohn A. (2013). South American Monogenoidea Parasites of Fishes, Amphibians and Reptiles.

[6] Kritsky D.C., Thatcher V.E., Kayton R.J. (1980). Five new species from South America with the proposal of *Tereancistrum* gen. n. and *Trinibaculum* gen. n. (Dactylogyridae: Ancyrocephalinae). Acta Amaz..

[7] Lizama M.L.A.P., Takemoto R.M., Pavanelli G.C. (2004). New species of *Tereancistrum* Kritsky, Thatcher & Kayton, 1980 (Monogenea: Dactylogyridae: Ancyrocephalinae) from the gills of *Prochilodus lineatus* (Osteichthyes: Prochilodontidae) from the upper Paraná River floodplain, Brazil. Syst. Parasitol..

[8] Cepeda P.B., Ceccarelli P.S., Luque J.L. (2012). A new species of *Tereancistrum* (Monogenea, Dactylogyridae) parasitic on *Prochilodus lineatus* (Valenciennes, 1837) (Characiformes) from Mogi Guaçu River, Brazil. Neotrop. Helminthol..

[9] Cohen S.C., Kohn A., Boeger W.A. (2012). Neotropical Monogenoidea. 57. Nine new species of Dactylogyridae (Monogenoidea) from the gill of *Salminus brasiliensis* (Characidae, Characiformes) from the Paraná river, State of Paraná, Brazil. Zootaxa.

[10] Karling L.C., Lopes L.P.C., Takemoto R.M., Pavanelli G.C. (2014). New species of *Tereancistrum* (Dactylogyridae) monogenean parasites of *Schizodon borellii* (Characiformes, Anostomidae) from Brazil, and emended diagnosis for *T. parvus*. Acta Sci. Biol. Sci..

[11] Zago A.C., Yamada F.H., Franceschini L., Bongiovani M.F., Yamada P.O.F., Silva R.J. (2017). A new species of *Tereancistrum* (Monogenea, Dactylogyridae) from the gills of three *Leporinus* species (Characiformes, Anostomidae) and a revised description of *Tereancistrum parvus*. An. Acad. Bras. Ciênc..

[12] Leite L.A.R., Pelegrini L.S., Azevedo R.K.D., Abdallah V.D. (2020). A new species of *Tereancistrum* (Monogenea: Dactylogyridae), parasite of *Prochilodus lineatus* (Characiformes: Prochilodontidae) from southeast Brazil. Braz. J. Vet. Parasitol..

[13] Hasuike W.T., Scorsim B., Arjona I.S., Amaral R.B., Damacena-Silva L., Araújo G.A., Bellay S., Oliveira A.V., Takemoto R.M. (2025). Morphology and molecular characterization of a new species of *Tereancistrum* parasite from the gills of *Brycon nattereri*. J. Helminthol..

[14] Mendoza-Palmero C.A., Blasco-Costa I., Scholz T. (2015). Molecular phylogeny of Neotropical monogeneans (Platyhelminthes: Monogenea) from catfishes (Siluriformes). Parasites Vectors.

[15] Gasques L.S., Graça R.J., Prioli S.M., Takemoto R.M., Prioli A.J. (2016). Molecular characterization of *Urocleidoides cuiabai* and *U. malabaricusi* (Monogenea: Dactylogyridae) from the trahira fish *Hoplias* aff. *malabaricus* in the Paraná River, Brazil. J. Helminthol..

[16] Acosta A.A., Mendoza-Palmero C.A., Silva R.J., Scholz T. (2019). A new genus and four new species of dactylogyrids (Monogenea), gill parasites of pimelodid catfishes (Siluriformes: Pimelodidae) in South America and the reassignment of *Urocleidoides megorchis* Mizelle et Kritsky, 1969. Folia Parasitol..

[17] Santos-Neto J.F., Domingues M.V. (2023). Integrative taxonomy of *Urocleidoides* spp. (Monogenoidea: Dactylogyridae) parasites of characiform and gymnotiform fishes from the coastal drainages of the Eastern Amazon, Brazil. J. Helminthol..

[18] Zago A.C., Yamada F.H., Yamada P.O.F., Franceschini L., Bongiovani M.F., Silva R.J. (2020). Seven new species of *Urocleidoides* (Monogenea: Dactylogyridae) from Brazilian fishes supported by morphological and molecular data. Parasitol. Res..

[19] Santos-Neto J.F., Domingues M.V. (2024). Integrative Taxonomy of *Urocleidoides* spp. (Monogenoidea, Dactylogyridae) Parasites of *Pseudanos trimaculatus* (Characiformes: Anostomidae) from Eastern Amazon, Brazil. Syst. Parasitol..

[20] Yamada P.O.F., Osaki-Pereira M.M., Acosta A.A., Ebert M.B., Silva R.J. (2024). Two new species of *Urocleidoides* (Monopisthocotyla: Dactylogyridae) parasitizing the gills of *Cyphocharax modestus* (Characiformes: Curimatidae) supported by morphological and molecular data. Diversity.

[21] Kritsky D.C., Thatcher V.E., Boeger W.A. (1986). Neotropical Monogenea. 8. Revision of *Urocleidoides* (Dactylogyridae, Ancyrocephalinae). P. Helm. Soc. Wash..

[22] Mizelle D., Klucka A.R. (1953). Studies on monogenetic trematodes. XVI. Dactylogyridae from Wisconsin fishes. Am. Midl. Nat..

[23] Kritsky D.C., Boeger W.A., Thatcher V.E. (1985). Neotropical Monogenea. 7. Parasites of the pirarucu, *Arapaima gigas* (Cuvier), with descriptions of two new species and redescription of *Dawestrema cycloancistrium* Price and Nowlin, 1967 (Dactylogyridae: Ancyrocephalinae). Proc. Biol. Soc. Wash..

[24] Mizelle J.D., Price C.E. (1963). Additional haptoral hooks in the genus Dactylogyrus. J. Parasitol..

[25] Bush A.O., Laferty K.D., Lotz J.M., Shostak A.W. (1997). Parasitolgy meets ecology on its own terms: Margolis revisited. J. Parasitol..

[26] Pleijel F., Jondelius U., Norlinder E., Nygren A., Oxelman B., Schander C., Sundberg P., Thollesson M. (2008). Phylogenies without roots? A plea for the use of vouchers in molecular phylogenetic studies. Mol. Phylogenet. Evol..

[27] Lockyer A.E., Olson P.D., Littlewood D.T.J. (2003). Utility of complete large and small subunit rRNA genes in resolving the phylogeny of the Neodermata (Platyhelminthes): Implications and a review of the cercomer theory. Biol. J. Linn. Soc..

[28] Yamada P.O.F., Müller M.I., Zago A.C., Yamada F.H., Ebert M.B., Franceschini L., Silva R.J. (2023). Three new species of *Jainus* (Monogenea: Dactylogyridae) parasitizing gills of Brazilian freshwater fishes supported by morphological and molecular data. Diversity.

[29] Kearse M., Moir R., Wilson A., Stones-Havas S., Cheung M., Sturrock S., Buxton S., Cooper A., Markowitz S., Duran C. (2012). Geneious Basic: An integrated and extendable desktop software platform for the organization and analysis of sequence data. Bioinformatics.

[30] Posada D. (2008). jModelTest: Phylogenetic model averaging. Mol. Biol. Evol..

[31] Ronquist F., Teslenko F.M., Van DerMark P., Ayres D.L., Darling A., Höhna S., Larget S.B., Liu L., Suchard M.A., Huelsenbeck J.P. (2012). MrBayes 3.2: Efficient Bayesian phylogenetic inference and model choice across a large model space. Syst. Biol..

[32] Miller M.A., Pfeiffer W., Schwartz T. Creating the CIPRES Science Gateway for inference of large phylogenetic trees. Proceedings of the 2010 Gateway Computing Environments Workshop (GCE).

[33] Guindon S., Gascuel O. (2003). A simple, fast, and accurate algorithm to estimate large phylogenies by maximum likelihood. Syst. Biol..

[34] Rambaut A. 2009—FigTree Version 1.3.1. Molecular Evolution, Phylogenetics and Epidemiology. Fig-Tree. http://tree.bio.ed.ac.uk/software/figtree.

[35] Tamura K., Stecher G., Kumar S. (2021). MEGA 11: Molecular Evolutionary Genetics Analysis Version 11. Mol. Biol. Evol..

[36] Kimura M. (1980). A simple method for estimating evolutionary rate of base substitutions through comparative studies of nucleotide sequences. J. Mol. Evol..

[37] Mizelle J.D. (1936). New species of trematodes from the gills of lllinois fishes. Am. Midl. Nat..

[38] Abdallah V.D., Azevedo R.K., Alves K.G.D., Camargo A.A., Vieira D.H.M.D., Silva R.J. (2016). The morphology of *Tereancistrum paranaensis* (Dactylogyridae) infecting *Schizodon intermedius*, with a key to the species. Neotrop. Helminthol..

[39] Stecher G., Tamura K., Kumar S. (2020). Molecular Evolutionary Genetics Analysis (MEGA) for macOS. Mol. Biol. Evol..

[40] Mendoza-Palmero C.A., Mendoza-Franco E.F., Acosta A.A., Scholz T. (2019). *Walteriella* n. g. (Monogenoidea: Dactylogyridae) from the gills of pimelodid catfishes (Siluriformes: Pimelodidae) from the Peruvian Amazonia based on morphological and molecular data. Syst. Parasitol..

[41] Zago A.C., Franceschini L., Müller M.I., Silva R.J. (2018). A new species of *Cacatuocotyle* (Monogenea, Dactylogyridae) parasitizing *Astyanax* spp. (Characiformes, Characidae) from Brazil, including molecular data and a key to species identification. Acta Parasitol..

[42] Acosta A.A., Scholz T., Blasco-Costa I., Alves P.V., Silva R.J. (2018). A new genus and two new species of dactylogyrid monogeneans from gills of Neotropical catfishes (Siluriformes: Doradidae and Loricariidae). Parasitol. Int..

[43] Franceschini L., Zago A.C., Müller M.I., Francisco C.J., Takemoto R.M., Silva R.J. (2018). Morphology and molecular characterization of *Demidospermus spirophallus* n. sp., *D. prolixus* n. sp. (Monogenea: Dactylogyridae) and a redescription of *D. anus* in siluriform catfish from Brazil. J. Helminthol..

[44] Zago A.C., Franceschini L., Abdallah V.D., Müller M.I., Azevedo R.K., Silva R.J. (2021). Morphological and molecular data of new species of *Characithecium* and *Diaphorocleidus* (Monogenea: Dactylogyridae) from Neotropical characid fishes. Parasit. Int..

[45] Acosta A.A., Franceschini L., Zago A.C., Scholz T., Silva R.J. (2017). Six new species of *Heteropriapulus* (Monogenea: Dactylogyridae) from South American fishes with an amended diagnosis to the genus. Zootaxa.

[46] Osaki-Pereira M.M., Narciso R.B., Vieira D.H.M.D., Müller M.I., Ebert M.B., Silva R.J. (2023). Molecular phylogeny of two *Rhinoxenus* species (Monogenea: Dactylogyridae) from the nasal cavities of serrasalmids (Characiformes: Serrasalmidae) from Brazil. Syst. Parasitol..

[47] Franceschini L., Acosta A.A., Zago A.C., Müller M.I., Silva R.J. (2020). *Trinigyrus* spp. (Monogenea: Dactylogyridae) from Brazilian catfishes: New species, molecular data and new morphological contributions to the genus. J. Helminthol..

[48] Oliveira G.S., Silva R.J., Vieira F.E.G., Acosta A.A. (2021). *Urocleidoides* spp. (Monogenea: Dactylogyridae) from the gills of *Parodon nasus* (Characiformes: Parodontidae) from a Brazilian stream with descriptions of two new species. Zootaxa.

[49] Wu X.Y., Zhu X.Q., Xie M.Q., Li A.X. (2006). The radiation of *Haliotrema* (Monogenea: Dactylogyridae: Ancyrocephalinae): Molecular evidence and explanation inferred from LSU rDNA sequences. Parasitology.

